# Effects of Excess Iron on the Retina: Insights From Clinical Cases and Animal Models of Iron Disorders

**DOI:** 10.3389/fnins.2021.794809

**Published:** 2022-02-03

**Authors:** Ali Shahandeh, Bang V. Bui, David I. Finkelstein, Christine T. O. Nguyen

**Affiliations:** ^1^Department of Optometry and Vision Sciences, Faculty of Medicine, Dentistry and Health Sciences, University of Melbourne, Parkville, VIC, Australia; ^2^Florey Department of Neuroscience and Mental Health, University of Melbourne, Parkville, VIC, Australia

**Keywords:** retina, iron, hemochromatosis, iron overload, retinal disorder

## Abstract

Iron plays an important role in a wide range of metabolic pathways that are important for neuronal health. Excessive levels of iron, however, can promote toxicity and cell death. An example of an iron overload disorder is hemochromatosis (HH) which is a genetic disorder of iron metabolism in which the body’s ability to regulate iron absorption is altered, resulting in iron build-up and injury in several organs. The retina was traditionally assumed to be protected from high levels of systemic iron overload by the blood-retina barrier. However, recent data shows that expression of genes that are associated with HH can disrupt retinal iron metabolism. Thus, the effects of iron overload on the retina have become an area of research interest, as excessively high levels of iron are implicated in several retinal disorders, most notably age–related macular degeneration. This review is an effort to highlight risk factors for excessive levels of systemic iron build-up in the retina and its potential impact on the eye health. Information is integrated across clinical and preclinical animal studies to provide insights into the effects of systemic iron loading on the retina.

## Introduction

Iron is essential for neuronal development and function (Lozoff et al., 2003). Iron plays key roles in neuronal metabolism, oxygen transport, oxidative phosphorylation, myelin production, and the synthesis of neurotransmitters, as reviewed in detail elsewhere (Crichton et al., 2011; Ward et al., 2014). However, abnormally high levels of intracellular iron, termed iron loading, can be neurotoxic and may lead to cellular injury. The exact mechanism of iron-induced neuronal degeneration is not yet fully understood; however, several mechanisms have been postulated. Perhaps, most well studied mechanism is the role of iron in mediating the formation of reactive oxygen species (ROS), a natural byproduct of Fenton reactions, which can contribute to tissue injury (Lloyd et al., 1997). In addition, high intracellular iron levels can affect the expression of several classes of genes including iron-dependent oxidative metabolism and the attendant oxidative stress genes (Casey et al., 1988; Ingrassia et al., 2019). Another suggested pathway through which errors in iron metabolism can be deleterious is ferroptosis, a recently identified form of cell death, which has been implicated in a number of central nervous system (CNS) diseases (Dixon et al., 2012; Guiney et al., 2017; Lane et al., 2018; Moreau et al., 2018; Masaldan et al., 2019).

To avoid the potential of iron toxicity, living organisms have developed homeostatic mechanisms, that regulate various proteins involved in iron import, storage, and export to maintain iron within a favorable physiological range. Most iron is tightly complexed with proteins or other ligands, which restrict its redox potential. However, excess iron can build-up in the body in several ways including early life exposure to iron feeding (Hare et al., 2015); normal aging (Zecca et al., 2004), eating meat (heme-iron intake) (Haider et al., 2018), and acute toxicity from blood transfusions (Harmatz et al., 2000; Cunningham et al., 2004). In contrast low iron vegan and vegetarian diets have lower accumulation of iron and increased risk of iron deficiency with associated fatigue and impaired learning and concentration symptoms (Desmond et al., 2021). The retinal photoreceptors are the most highly energetically demanding tissue per weight in the whole body (Wong-Riley, 2010; Country, 2017), and photoreceptors are mitochondria-rich cells. The primary function of mitochondria is to generate energy in the form of adenosine triphosphate (ATP). Due to a lack of protective histones and proximity to the inner mitochondrial membrane, where oxidants are formed, mitochondrial DNA is vulnerable to oxidative damage (Gao et al., 2009; Lin et al., 2011). Iron-mediated damage to mitochondria is believed to be through various pathways including altered mitochondrial uptake of other metal ions (Jouihan et al., 2008), ferroptosis (Battaglia et al., 2020; Li et al., 2020) and oxidative damage leading to decreased synthesis of respiratory chain subunits encoded by the mitochondrial genome and further declines in cellular respiration. Mitochondrial iron homeostasis is crucial for normal physiology of the retina and any dysregulation in balance of the redox system in mitochondria has been associated with age-related retinal pathophysiology (Jarrett et al., 2008; Barot et al., 2011).

However, unlike the brain where iron-related pathology has received significant attention (Wessling-Resnick, 2017; Daglas and Adlard, 2018; D’Mello and Kindy, 2020), less is known about retinal susceptibility to iron loading. An improved understanding of iron’s role in retinal health and disease conditions will guide the development of therapies for patients with hemochromatosis and possibly other iron overload conditions. We mainly focus on retinal structural and functional changes in clinical cases and preclinical models. This is particularly important because currently there is no routine clinical visual check–up for high-risk demographic such as patients with hemochromatosis or even people with high levels of dietary iron intake which may have been at risk of retinal iron accumulation and disease-related sequelae. Improving prognosis in people at risk, as some changes may be more easily attenuated or reversed than others are critical and subsequently treatments could be commenced before irreversible damage to the tissue occurs.

## Hemochromatosis

Iron build-up in the human body can arise as a result of genetic anomalies, one such example is hemochromatosis (HH) (Feder et al., 1996; Pietrangelo, 2003). HH is an autosomal recessive disorder characterized by increased iron absorption (Andrews, 1999), leading to excessive tissue iron deposition. A number of gene mutations have been found to underlie this phenotype as summarized in Table 1.

**TABLE 1:** Summary of hemochromatosis phenotypes with their corresponding genes and clinical manifestations^a^.

Types		Main clinical manifestations	Inheritance
Type 1	*HFE* *	Late onset, >30 years old, mild to severe hepatomegaly, elevated aminotransferase levels, arthralgia, arthritis	Recessive
Type 2	Hemojuvelin (*H*JV)/ Hepcidin (*HAMP*)	Early onset, <30 years old, severe cardiomyopathy, arrhythmia, diabetes, hypogonadism	Recessive
Type 3	*TFR2*	Late onset, >30 years old, mild to severe hepatomegaly, elevated aminotransferase levels	Recessive
Type 4	Ferroportin (*SLC40A1*)	Late onset, >40 years old, mild hepatomegaly, elevated aminotransferase levels, arthralgia, arthritis	Dominant

*^a^Pietrangelo, 2004; Brissot and de Bels, 2006; Andrews, 2008; Fix and Kowdley, 2008; Powell et al., 2016.*

*Variation in this gene including C282Y, H63D, and S65C are associated with increasing risk of neurodegenerative disorders particularly Parkinson’s disease.

There are four main types of HH which have been identified based on their underlying genetic mutations. Type 1 forms the majority of HH cases and is attributed to homozygosity for a single nucleotide polymorphism (SNP) in the *HFE* gene located on chromosome 6p. This genetic variation causes the substitution of a cysteine with a tyrosine residue at amino acid 282 (C282Y) (Feder et al., 1996). The C282Y SNP has a carrier frequency of 10–20%, with 0.3–0.7% homozygosity in populations of northern European ancestry (Beutler et al., 2002; Allen, 2008). In non-Caucasian populations, however, this SNP is reported to have a low allele frequency (Merryweather-Clarke et al., 1997; Cullen et al., 1998).

Another allelic variant of the *HFE* gene involving the substitution of an aspartic acid with a histidine (H63D), has an even higher allele frequency that C282Y (Gochee et al., 2002; Adams, 2005). However, it generally does not appear to have considerable effects on iron status, even in the homozygous state. Inheritance of a dual polymorphism (H63D and C282Y) as a compound heterozygous genotype, however, can cause clinical hemochromatosis (Beutler et al., 1996; Jouanolle et al., 1997). *HFE* – associated hemochromatosis can also be due to a serine – to – cysteine substitution at amino acid position 65 (S65C) (Mura et al., 1999). This variation in the *HFE* gene is associated with the mild form of hemochromatosis and accounts for 7.8% of HH cases that were neither C282Y nor H63D substitutions (Mura et al., 1999).

Type 2 HH is due to mutations in the hemojuvelin gene (*HJV*) or the gene encoding hepcidin (*HAMP*); accounts for a very severe form of iron loading where iron accumulates rapidly in early postnatal life (Papanikolaou et al., 2004).

Mutations in the *TFR2* gene causes Type 3 HH in which homozygosity for the Y250X nonsense mutation in the *TFR2* gene is reported to be the most common cause (Camaschella et al., 2000). This mutation induces excessive iron loading with a similar phenotype to Type 1 HH, although patients exhibit greater variation in the severity of symptoms (Olynyk et al., 2008). Patients with mutations in both *TFR2* and *HFE* are reported to have a severe iron loading phenotype (Pietrangelo et al., 2005; Rueda Adel et al., 2011; Del-Castillo-Rueda et al., 2012). The common feature of HH Types 1 to 3 is reduction of hepcidin expression which provides a possible mechanistic insight into build-up of iron in tissues (Brissot et al., 2019; Piperno et al., 2020).

Considering the importance of the hepcidin regulatory pathway in influencing body iron homeostasis, any mutation in the *SLC40A1* gene which encodes ferroportin; the molecular target for hepcidin results in Type 4 HH (Camaschella et al., 2000; Montosi et al., 2001; Callebaut et al., 2014). In this type of HH, mutation in *SLC40A1* gene either restricts the binding of hepcidin to ferroportin (Nemeth et al., 2004) or affects the ability of ferroportin to transport iron (Détivaud et al., 2013).

Most forms of hemochromatosis cause injury in peripheral tissues (heart and liver), however, it should be noted that the principal variants of the *HFE* gene can affect brain iron homeostasis and is considered as a risk factor for neurodegenerative disorders, particularly Parkinson’s disease (Dekker et al., 2003; Guerreiro et al., 2006). In addition, hematochromatosis associated genes are expressed in the retina in a cell type specific manner (Gnana-Prakasam et al., 2008), which means retinal iron homeostasis may be disrupted in specific retinal cells by systemic iron loading conditions.

## The Mammalian Retina

**FIGURE 1:**
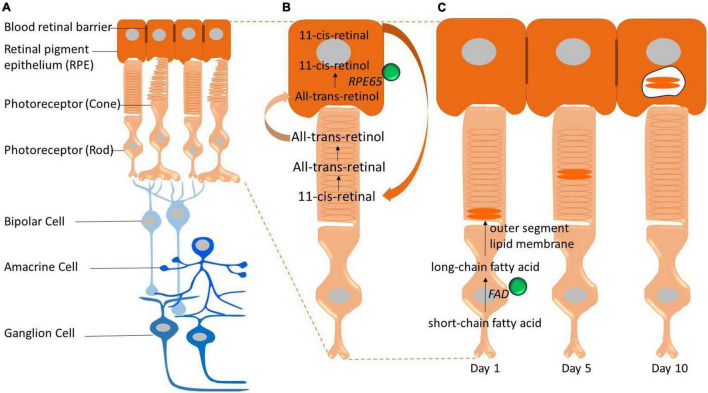
Schematic representation of the retina and key pathways that involve iron (iron represented by green spheres). **(A)** The neural retina is isolated from the bloodstream (choroidal vessels) by the blood–retinal barrier. The outer blood–retinal–barrier is formed by tight junctions located between the retinal pigment epithelium (RPE). Photoreceptors convert light into electrical signals through the visual cycle and these electrical signals are communicated through the retina *via* the bipolar and ganglion cells. **(B)** Iron is required for the visual cycle. Regeneration of 11-cis-retinal, the chromophore of visual pigments in photoreceptor cells, is essential for vision and depends on an earlier reaction catalyzed by the iron-containing isomerase, retinal pigment epithelium-specific 65 kDa protein (RPE65). **(C)** Photoreceptor outer segments (rods and cones) are constantly synthesized and shed. This process is highly dependent on fatty acid desaturase (FAD) enzyme which is iron dependent to synthesize new membrane lipids.

The mammalian retina is an integral part of the CNS; capturing light energy (photons) and transforming it into a chemical signal to be passed onto the brain *via* electrical impulses. The retina has a multi–layered structure (see Figures 1A,B) composed of five main neuronal classes and supporting glia. Traditionally, it has been assumed that the retina is protected from systemic iron loading by the blood–retinal barrier, however, increasing evidence suggests that the blood–retinal barrier is unable to shield the retina against morbid levels of serum iron (Zhao et al., 2014). This is potentially problematic for the retina, given it is highly energetically demanding (Wong-Riley, 2010; Country, 2017), making it more likely vulnerable to metabolic and oxidative stress related to iron-loading. The potential for injury from excessive iron loading in the retina has become an area of research interest, particularly in conditions where the integrity of the blood–retina barrier is compromised. Indeed, studies have highlighted interactions between aging and iron homeostasis as potential pathological mediators for a number of retinal disorders including age-related macular degeneration (Dunaief, 2006; Biesemeier et al., 2015; Song et al., 2016; Čolak et al., 2018).

## Iron Metabolism and Retinal Iron Homeostasis

Dietary iron is the exclusive source of iron for mammals which consists of heme iron (e.g., meat or offal products) and non-heme iron (e.g., legumes, nuts, and seeds) sources (Bjorn-Rasmussen et al., 1974). Iron cannot be actively excreted through normal pathways such as the intestine or kidney and only small levels of iron can be discarded through sloughing of mucosal cells and desquamation, menstruation and other blood loss (Andrews, 1999; Miret et al., 2003; Kohgo et al., 2008). One of the earliest experiments to show a lack of execratory routs for iron was conducted by Widdowson and McCance (1937). The author orally administrated 1000 mg of ferric ammonium citrate per day to four volunteers over 36–46 days, and found that urinary and fecal output contained only trace amounts of unabsorbed iron (Widdowson and McCance, 1937). This work contributed to the current understanding that iron metabolism is a semi-closed system, with low levels of excretion (Kohgo et al., 2008). This closed system means that non-biodegradable iron can build-up in tissues over time, as reviewed elsewhere (Hahn et al., 2006, 2009; Hare et al., 2015, 2017; Shu and Dunaief, 2018). As iron is rare in our environment the body has adopted to store iron. In an iron replete modern diet, the abundance of iron is potentially contributing to health issues. Therefore, age-related accumulation of iron and its adverse effects may be a contributor to increased risk of neurodegeneration with age.

Dietary iron is chiefly absorbed in the upper part of the intestine through the apical side of the enterocyte brush border (Fuqua et al., 2012). After absorption across the gut, iron forms a complex, most notably with the iron carrier protein transferrin (TF), which chaperones iron in the circulation (Andrews, 1999; Meynard et al., 2014). A transferrin molecule can bind two molecules of ferric (Fe^3+^) iron and is thought to be the main source of iron delivery to neurons in the CNS (Chua et al., 2007).

The specific locations of iron handling proteins in the retina as summarized in Table 2 may provide clues as to functions that may be impacted by anomalies in iron metabolism and dysregulation. In the retina, transferrin is found in RPE cells, but it is also detectable in the neural retina including photoreceptor inner and outer segments, suggesting that iron is trafficked in the retina itself. Iron uptake from the choroidal circulation into the RPE cells is mediated by transferrin receptors 1 (TFR1), which has been found on basolateral and apical surfaces of RPE cells, suggesting that there is bi-directional iron movement across the RPE cells depending on the iron status of the retina (Yefimova et al., 2000; He et al., 2007). TFR1 has also been colocalized to the ganglion cell layer, inner nuclear layer, outer plexiform layer, and inner segment of photoreceptors (Yefimova et al., 2000; He et al., 2007).

**TABLE 2:** Summary of the major iron-related proteins found in the retina.

	Protein	Function	Expression in retina
Iron import	Transferrin receptor 1 (TFR1)	Iron uptake	Ganglion cell layer, inner nuclear layer, outer plexiform layer, photoreceptor inner segment, RPE basolateral membrane, and choroid (Yefimova et al., 2000)
	Transferrin receptor 2 (TFR2)	Iron uptake	RPE (basolateral membrane) (Martin et al., 2006)
	Divalent metal transporter-1 (DMT-1)	Iron uptake	Rod bipolar cell bodies and axon termini, horizontal cell bodies, and photoreceptor inner segments (He et al., 2007)
Iron export	Ferroportin (SLC40A1)	Iron export	RPE, photoreceptor inner segments, inner and outer plexiform layers, and ganglion cell layer (Hahn et al., 2004a)
Iron storage	Ferritin (Ft)	Storage; anti-oxidant activity in association with p53 (Lee et al., 2009); regulator of angiogenesis (Coffman et al., 2009); receptor for hepcidin (Nemeth et al., 2004)	Photoreceptor inner segments, RPE, choroid, inner nuclear layer, and ganglion cell layer (He et al., 2007)
Iron regulatory	Hepcidin (HAMP)	Internalization of iron in association with ferroportin	Müller cells, photoreceptor cells, and RPE (Gnana-Prakasam et al., 2008; Hadziahmetovic et al., 2011a)
	Ceruloplasmin (CP)	Catalyzing the oxidization of Fe^2+^ to Fe^3+^ (ferroxidase)	Müller glia and RPE(Chen et al., 2003; Hahn et al., 2004b)
	Hephaestin (HEPH)	Catalyzing the oxidization of Fe^2+^ to Fe^3+^ (ferroxidase)	Müller glia and RPE (Hahn et al., 2004b)
	HFE	HFE in association with TFR2 regulates hepcidin (Wallace et al., 2009)	Restricted exclusively to basolateral membrane of RPE (Martin et al., 2006)
	Hemojuvelin (HJV)	Induces the expression of hepcidin (Babitt et al., 2006)	RPE (apical membrane facing neural retina), Müller glia, photoreceptors, and ganglion cells (Gnana-Prakasam et al., 2009b)

Although TFR1 is the predominant protein responsible for cellular iron uptake, another homologous protein called transferrin receptors 2 (TFR2) also has a cooperative role in mediating retinal cellular iron uptake (Kawabata et al., 1999). The expression of TFR2 appears to be primarily in the RPE (Martin et al., 2006). TFR2 also serves as part of a feedback mechanism *via* its role as a membrane protein for serum iron levels. TFR2 is postulated to activate a downstream signaling pathway that leads to increase the iron-regulatory protein hepcidin. Increased levels of hepcidin, tightly influences intestinal iron absorption and macrophage iron recycling, helping to lower serum iron (Goswami and Andrews, 2006; Schmidt et al., 2008).

In addition, there is a non-transferrin-bound iron protein called divalent metal transporter-1 (DMT-1), which can contribute to transportation of ferrous iron across the plasma membrane or in some cases out of endosomal compartments (Gunshin et al., 1997). DMT-1 has been colocalized to rod bipolar cell bodies and axon termini, horizontal cell bodies and photoreceptor inner segments (He et al., 2007; Song and Dunaief, 2013).

Once iron is taken up into cells, it is primarily stored in ferritin, a large hollow symmetrical protein which is able to sequester around 4500 atoms of iron (Harrison and Arosio, 1996; Koorts and Viljoen, 2007; Arosio et al., 2009). In addition to the choroid and RPE, within the neural retina the regions with highest levels of iron and ferritin are found in the inner segment of photoreceptors, inner nuclear layer and the ganglion layer (Yefimova et al., 2000; He et al., 2007).

Iron efflux is regulated through a transmembrane protein called ferroportin. It is also known as an iron-regulated transporter 1 or solute carrier family 40 member 1 which exports iron from cells in the form of ferrous (Fe^2+^) iron. Since ferrous iron must be oxidized to its ferric state to be regulated by systemic transferrin, ferroportin functions in conjunction with two extracellular multicopper ferroxidases, ceruloplasmin and hephaestin (Harris et al., 1999; Vulpe et al., 1999; Jeong and David, 2003; Ayton et al., 2013; McCarthy and Kosman, 2013). Ferroportin is found on most cells including retinal neurons and glia (Abboud and Haile, 2000; Donovan et al., 2000, 2005; Jeong and David, 2003; Hahn et al., 2004a). Ferroxidase enzymes including ceruloplasmin and hephaestin are also present in the retina, specifically in Müller glia and retinal pigment epithelial cells (Chen et al., 2003; Hahn et al., 2004b).

Another iron-regulatory protein which plays a crucial role in the maintenance of retinal iron homeostasis is hepcidin (Nicolas et al., 2001; Pigeon et al., 2001). Hepcidin is the main peptide hormone that regulates systemic iron. Hepcidin is primarily expressed by the liver and secreted into the bloodstream, and expression of hepcidin in retinal Müller cells, photoreceptor cells and RPE cells has been reported (Gnana-Prakasam et al., 2008; Hadziahmetovic et al., 2011a). Hepcidin regulates iron homeostasis by binding and antagonizing the only known mammalian iron exporter, ferroportin, leading to reduced cellular iron export thus limiting the amount of iron found in serum or extracellular fluid (Nemeth et al., 2004). Hepcidin also plays an important role in inhibiting the intestinal iron absorption (Ganz, 2005). Finally, hepcidin promotes sequestration of iron by macrophages which further reduce serum iron levels (Abreu et al., 2018).

## Role of Iron in Retinal Physiology

Iron is one of the most abundant metals in the retina (Ugarte et al., 2012), critical for retinal function *via* its role as an integral component of key retinal enzymes (Lohmann, 1964; Yau and Baylor, 1989; Yefimova et al., 2000; Moiseyev et al., 2006; Chen et al., 2009). The characterization of retinal iron content through histochemical approaches showed a heterogeneous distribution, with iron largely found at the choroid, RPE and the photoreceptors layer (Yefimova et al., 2000; Song and Dunaief, 2013). Similarly, iron colocalization at the RPE, choroid and to a lesser degree at the neural retain was confirmed using laser ablation-inductively coupled plasma-mass spectrometry (LA-ICP-MS) which is a sensitive technique that offers a fast and precise spatially resolved measurement of elements *in situ* at trace and ultra-trace levels (Pamphlett et al., 2020).

In the context of visual function, iron serves as a cofactor for the RPE-specific 65 kDa protein (RPE65), an enzyme required for the retinoid metabolic pathway, which is critical for the maintenance of light capturing chromophore levels in photoreceptors and thus phototransduction and visual adaption (Redmond et al., 2005; Moiseyev et al., 2006). The isomerohydrolase activity of RPE65 relies on the presence of metal ions (see Figure 1B). Iron is central to this process, as evidenced by findings that mutations in RPE65 iron coordinating result in a range of retinal dystrophies including Leber congenital amaurosis and some forms of retinitis pigmentosa (Thompson et al., 2000; Simovich et al., 2001; Redmond et al., 2005; Bereta et al., 2008).

In addition, iron is an essential cofactor of fatty acid desaturases enzymes which play an important role in photoreceptor outer segment disc biogenesis (Figure 1C; Shichi, 1969; Song and Dunaief, 2013); whereby old photoreceptor discs containing phototransduction proteins are completely replaced by new ones over the course of about 10 days (Young, 1967; Young and Bok, 1969; Chuang et al., 2007). In addition to the above retina specific role for iron, like the CNS, the retina expresses several iron-dependent enzymes including tyrosine hydroxylase (Ramsey et al., 1996), phenylalanine hydroxylase (Gottschall et al., 1982), and tryptophan hydroxylase (Kuhn et al., 1980) that are involved in the biosynthesis and regulation of neurotransmitters such as dopamine, serotonin, and melatonin (Kaushik et al., 2007). Therefore it is not surprising that the retina expresses a wide array of enzymes that utilize iron as a cofactor, including those involved in energy production; mitochondrial ferredoxins, Redfearn and King (1964) cytochromes (Slater, 1949), and aconitase (Dickman and Cloutier, 1950).

## Retinal Changes in Hemochromatosis

One way to consider the importance of iron to the retina and visual function is to examine what happens in those people who have an iron loading disorder. However, assessment of retinal changes in hemochromatosis patients remain limited. One of the earliest reports on the ocular manifestations of high levels of retinal iron loading in hemochromatosis patients was described by Maddox (1933). The author reported that there was normal visual acuity without overt retinal pathology in four cases (Maddox, 1933). Almost 40 years after Maddox, further reports in those with hemochromatosis showed iron accumulation in the peripapillary (around the optic nerve) retinal pigment epithelium, ciliary epithelium (where aqueous humor is produced), corneal epithelium, and sclera (Davies et al., 1972; Roth and Foos, 1972). Drusen-like deposits, which are the prominent clinical feature of age-related macular degeneration, were also detected in these patients (Roth and Foos, 1972). In a more recent study, Menghini et al. (2018) examined 49 patients (∼15 years after diagnosis, average age at examination 60 years) with hemochromatosis and examined the retina *in vivo* using spectral domain optical coherence tomography, as well as short wavelength autofluorescence and color fundus images. Their results showed no retinal changes such as drusen, RPE alterations, or increased lipofuscin in any of the images.

In contrast, other case studies that examine histopathology and retinal function *via* electroretinography have indicated the possibility of retinal changes with hemochromatosis. Retinal abnormalities have been reported in a 49-year-old hemochromatosis patient with homozygosity for the C282Y polymorphism in the *HFE* gene. The patient was characterized by progressive loss of visual function assessed by full – field electroretinography (ERG) and abnormal RPE changes using retinal imaging (Zerbib et al., 2015). Electroretinographic examination at the initial visit revealed a 50% reduction in the rod response and no change in the cone response. Follow-up examinations 6-month later suggested that in addition to a diminished rod response there was a progression of cone dysfunction, with a 50% reduction in the cone response (Zerbib et al., 2015).

Recently, another case of ocular involvement in hereditary hemochromatosis was reported in a 39-year-old patient with a homozygous C282Y mutation in *HFE* gene, without any other mutations associated with inherited retinal dystrophies. The patient reported bilateral progressive blurry vision and recent onset photopsia and headaches. Electroretinograms demonstrated generalized rod and cone dysfunction with some central preservation of waveforms. There appeared to be no retinopathy progression at 6 month follow-up, after the patient had undergone treatment to normalize his iron levels (Bellsmith et al., 2020).

There remain limited studies exploring the relationship between the systemic iron loading disorders and retinal changes. The heterogeneity in findings between the few case reports and Menghini et al.’s (2018) study could derive from the methodologies employed, for example, electroretinography can be a more sensitive measure of temporary retinal dysfunction than optical coherence tomography, which may only detect more significant structural changes associated with longer term retina injury (Ledolter et al., 2015).

## Animal Models of Iron Disorders of the Retina

To gain further insight, preclinical studies have sought to examine in animal models the impact of systemic iron loading on retinal health. Broadly, three types of animal models have been developed including local administration of exogenous iron (e.g., intravitreal injection), oral delivery of iron (dietary iron supplementation), and genetically modified iron loading models.

It should be noted during the earliest stages of neonatal development when mammals nurse on their mother’s milk, the blood–brain barrier is open to iron accumulation (Dwork, 1995; Kaur et al., 2007; Billings et al., 2016). Subsequently the blood–brain barrier becomes fully formed in early life and only then iron is acquired selectively through iron receptors proteins that mediate iron intake.

One of the earliest animal models of local administration of exogenous iron was conducted by Declercq et al. (1977). They examined the consequences of excess iron on retinal function and histopathology in an animal model of siderosis. Ten days after intravitreal injection of a solid iron foreign body into the rabbit eye, they reported reductions in both a- (photoreceptor) and b-wave (ON bipolar cells) amplitudes in conjunction with degeneration of the outer nuclear layer and RPE (Declercq et al., 1977). Subsequently, Doly et al. (1984) showed that intravitreal injection of a solution containing Fe^2+^ ascorbate into the rat’s eyes selectively attenuated the b-wave in isolated retinal recordings (Doly et al., 1984). A similar study showed intravitreal injection of ferrous sulfate (FeSO_4_) into mouse’s eyes caused a reduction in the b-wave amplitude (Rogers et al., 2007). These data show that high levels of iron appear to reduce outer and middle retinal function.

The effects of high dietary iron were studied on 2-month-old mice by supplementing mice with 2% iron carbonyl for 3 or 10 months. Compared with a normal diet, dietary iron supplementation changed retinal mRNA levels of iron-responsive genes indicating a modest increase in iron in the RPE. However, even by 18 months of age there was little evidence of increased retinal iron and no retinal degeneration (Bhoiwala et al., 2015). The authors suggested that high levels of dietary iron can only modestly increase the level of iron deposition in the mouse RPE without causing RPE or retinal degeneration, which means the blood–retinal barrier is able to regulate iron levels (Bhoiwala et al., 2015).

The effects of acute systemic iron loading *via* intravenous injection were investigated by Song et al. (2016). Their study involved two cohorts of C57BL/6J male mice including a group of 2 months old mice administrated a weekly dose of 1.2 mg iron-sucrose for a total of 12 injections. These animals were examined at 12 months of age. The second cohort of mice at 10 weeks of age were subjected to two intravenous injections either with iron-sucrose or sucrose alone and euthanized 24 h after the last injection. Their results showed iron accumulated chiefly at the RPE and choroid in chronic exposure. Additionally, histological features similar to AMD such as Bruch’s membrane thickening, RPE hypertrophy and vacuolization were noted in these mice. The second acute loading group displayed increased levels of ferritin mRNA and reduction of transferrin receptor mRNA within the RPE (Song et al., 2016).

Consistent with susceptibility of the choroid, RPE and outer retina, Song et al. (2016) reported a clinical case of a 43-year-old anemic patient who developed numerous retinal drusen, the hallmark of AMD, within 11 months of intravenous iron therapy. The patient who had no prior ocular history had undergone infusion of 300 mg of iron-sucrose saline over 2 h three times a week. As the treatment progressed the patient noted delayed adaptation to low-light levels and difficulty reading at night. The patient’s DNA was tested and revealed a high genetic risk for AMD. They concluded that iron therapy may have the potential to induce or exacerbate a form of retinal degeneration that shares features with AMD (Song et al., 2016).

It should be noted that the iron regulatory hormone, hepcidin is expressed in the neurosensory retina and may limit iron influx into the inner retina which would be another mechanism to protect the inner retina against high levels of iron (Baumann et al., 2019). Therefore, Shu et al. (2019) designed a study in which retina-specific hepcidin knockout mice were exposed to high systemic iron levels through daily intraperitoneal injection of 10 mg iron dextran for 5 days each week. Their results showed that while they could identify accumulation of high levels of iron in the RPE and retinal vascular endothelial cells of hepcidin knockout mice, there was no change in iron status of neurosensory retina. They reported no alterations in the retina–blood barrier and no signs of retinal degeneration. They concluded that the retinal–blood barrier can protect neurosensory retina from high doses of exogenously administered iron independent of retinal hepcidin production (Shu et al., 2019).

All of the studies analyzing the effects of acute exogenous iron-loading on retina showing similar patterns of iron distribution with RPE and choroid being the main sites of iron accumulation. However, the pathological effects of iron on retina seemed to be varied amongst these studies. Thus further studies of defective retinal iron homeostasis using both functional and histological outcome measures in genetically modified animal models of chronic iron loading could potentially provide a more comprehensive understanding of iron-induced retinal pathology.

### Retinal Abnormalities in Genetically – Modified Animal Models of Systemic Iron Loading

Genetically modified animal models of chronic iron loading have been developed to recapitulate human iron loading diseases. One of the earliest mouse models of systemic iron loading was developed by disruption of a single ferroxidase, either *Cp* or *Heph*. A study has shown that the *Cp* knock-out mice at 18 months old showed increased retinal iron levels and degeneration of the retina (Patel et al., 2002) in alignment with aceruloplasminemia patients who have mutations in Cp (Wolkow et al., 2011). In contrast, other animal studies which selectively delete either *Cp* or *Heph* did not manifest any retinal iron loading or related pathology even at 2 years of age (Hahn et al., 2004b), suggesting that one ferroxidase can compensate for the functional loss of the other (Jiang et al., 2016). However, simultaneous disruption of *Cp* and *Heph* in double knockout mice led to substantial retinal iron build-up (more than 2.5-fold) at 6-month of age compared to wild-type mice (Hahn et al., 2004b; Hadziahmetovic et al., 2008). Double knockout mice showed photoreceptor degeneration, sub-retinal neovascularization and RPE hypertrophy, hyperplasia by 9 months of age (Hahn et al., 2004b). Retinal degeneration in these mice was progressive, becoming more severe by 12–13 months of age (Hadziahmetovic et al., 2008).

Another mouse model of systemic iron loading was developed by Wolkow et al. (2012). Their murine model combined systemic ceruloplasmin deletion with RPE-specific hephaestin knockout resulted in iron accumulation in the RPE by 3 months of age, which was accompanied with a range of pathological features. In contrast, photoreceptor-specific deletion of hephaestin had no substantial effect on iron levels in the neural retina, even with concomitant systemic deletion of ceruloplasmin (Wolkow et al., 2012). Together, these results indicate that hephaestin has a cell-autonomous role in the RPE.

A further mouse model was generated by a single nucleotide (G to A) switch of the transferrin gene to produce hypotransferrinemic *Hpx^–/–^* mice (Trenor et al., 2000). Homozygous *Hpx^–/–^* mice have no transferrin and injection of human transferrin is required for survival, but even then, only a minority of mice survive up to 2 months of age (Lederman et al., 2012). Despite administration of exogenous human transferrin, the amount of transferrin in the retina of *Hpx^–/–^* mouse decreased to 61% of wild-type levels (Dickinson and Connor, 1995). Interestingly, the total iron content in the retina was not different between *Hpx^–/–^* and wild-type mice. Although gross retinal structure appeared to be normal in *Hpx^–/–^* mouse, assessment of retinal function showed attenuation of scotopic and photopic electroretinogram amplitudes compared to wild-type mice at 1 and 2 months of age (Lederman et al., 2012).

Finally, abnormal systemic iron loading has also been modeled by disruption of the bone morphogenetic protein 6 (*Bmp6*) gene, a key iron regulator gene that acts through hepcidin. In *Bmp6* knockout mice (*Bmp6^–/–^*) high levels of iron are found in the neural retina of male *Bmp6^–/–^* mice (fourfold compared to wild-type male mice) (Hadziahmetovic et al., 2011c). Male *Bmp6^–/–^* mice showed severe retinal pathology by ∼10 months of age, including RPE hypertrophy and hyperplasia, loss of overlying photoreceptors and outer nuclear layer thinning. It is of interest that male *Bmp6^–/–^* mice had almost 20-fold more iron in their RPE/choroid compared to female *Bmp6^–/–^* mice. Female *Bmp6^–/–^* mice showed no evidence of increased retinal iron loading and normal retinal morphology. The sexual dimorphism of iron effects in *Bmp6^–/–^* mice was studied by castration and ovariectomy. Higher levels of liver hepcidin was found in castrated *Bmp6^–/–^* males relative to non-castrated *Bmp6^–/–^* mice, while there was no evidence of abnormal hepcidin expression in ovariectomized females. The authors argued that higher iron accumulation in *Bmp6^–/–^* males may have arisen through additional suppressive effects of testosterone on hepcidin synthesis (Latour et al., 2014). These studies highlight that along with age, gender can affect levels of retinal iron loading in the retina (Hahn et al., 2006).

Whilst providing important insight, the genetic mouse models of iron dyshomeostasis introduced thus far are not genetically analogous to human hemochromatosis. Thus, further studies of mouse models with disruption of genes that predispose to heredity hemochromatosis could offer more clinical relevance.

### Retinal Abnormalities in Genetically – Modified Animal Models of Hemochromatosis

In addition to the above models, preclinical studies have sought to disrupt genes that predispose to heredity hemochromatosis in humans. The expression of hemochromatosis-related genes in mouse retina (Martin et al., 2006) makes it a useful animal model to address the possibility of iron-induced retinal injury.

Iacovelli et al. (2009) hypothesized that the disruption of ferroportin (Type 4 hemochromatosis) within the retina could lead to aberrant iron accumulation during both development and adulthood in mammals. They generated a polycythemia (high concentration of red blood cells) mouse (Pcm) model, and the results showed that up to 7 weeks of age there was no difference between Pcm and wild-type mice. However, at 12 months of age there were signs of retinal degeneration and morphological changes in aging Pcm mice. Unfortunately, retinal iron levels were not reported for Pcm mice at 1 year of age (Iacovelli et al., 2009).

The hepcidin knockout mouse (*Hamp^–/–^*) is another model of hemochromatosis (Type 2) that show age-dependent increases in retinal iron in the RPE/choroid and neural retina (Hadziahmetovic et al., 2011a). Retinal structure was normal at 3 months of age in *Hamp^–/–^* mice. Mild focal abnormalities became apparent by 9 months and by 18 months pathology was severe including RPE hyperplasia, accumulation of lipofuscin-like material in the RPE, loss of photoreceptor outer segments and subretinal neovascularization (Hadziahmetovic et al., 2011a). Histochemical analysis of retinal iron levels (Perls’ staining) showed that the RPE and non-pigmented ciliary epithelium are the primary sites of retinal iron accumulation in *Hamp^–/–^* mice older than 9-month. *Hamp^–/–^* mice also showed changes in retinal iron-related genes and proteins, including reduced *Tfr1* mRNA levels, and increased retinal L-ferritin protein levels at 4 months of age (Hadziahmetovic et al., 2011a). It is noticeable that despite progressive retinal iron accumulation in *Hamp^–/–^* mice and previously described *Cp* and *Heph* double knockout mice, there is a down-regulation in transferrin-bound iron import proteins in the retina of these models. This contradiction was resolved by Sterling et al. (2017) showing upregulation of non-transferrin bound iron import proteins in these two mouse models of retinal iron accumulation.

Another mouse model of hemochromatosis (Type 2) is hemojuvelin knockout (*Hjv^–/–^*) mice. This animal model showed early systemic iron loading in particular in the liver, pancreas and heart by two and a half months of age (Niederkofler et al., 2005), with little evidence of retina changes at this age. *Hjv^–/–^* mice showed retinal degeneration at 18 months of age as assessed by counting cell bodies in retinal cross sections (Gnana-Prakasam et al., 2012).

Finally, a mouse model of hemochromatosis Type 1, which is one of the most prevalent mutations seen in human is the *Hfe* knockout mouse (*Hfe^–/–^*). This mouse model had normal retinas at 6 months of age. Assessment at 18 months of age revealed severe retinal abnormalities including loss of ganglion cells, uneven distribution of nuclei in the inner and outer nuclear layers, widespread hypertrophy of RPE cells and focal areas of RPE hyperplasia (Gnana-Prakasam et al., 2009a). In addition, *Hfe^–/–^* mice showed changes in iron – related proteins including increased levels of both H-ferritin and L-ferritin in photoreceptors, outer and inner plexiform layers and within the RPE, in addition to reduced *Tfr1* mRNA levels (Gnana-Prakasam et al., 2009b).

### Summary of Animal Studies on Iron and Retina

The pathologic findings in animal models shows that too much iron in the eye leads to dysfunction of photoreceptors and downstream consequences on the inner retina. Under normal conditions it seems that the regulatory system including the blood–retinal barrier protects the retina from systemic dietary iron loading. However, high levels of serum iron can eventually damage the RPE, which then reduces support to photoreceptors. Additionally, disruption to genes associated with iron–regulation leads to iron build-up, particularly the RPE and photoreceptors, resulting in progressive photoreceptor and downstream inner retinal degeneration. There is some evidence that rod photoreceptor dysfunction precedes cone photoreceptor changes with iron accumulation in the RPE. The amount of retinal injury and rate of progression associated with systemic iron loading appears to be varied depending on the specific regulatory protein involved, in addition to the age and sex of the mice (Hahn et al., 2009). While there is an increasing interest in incorporating electroretinography and *in vivo* imaging, as well as gene expression changes in retinal studies in animal models of iron loading (Liu et al., 2021), the number of such studies are still limited. More comprehensive time course studies, particularly using complementary tools such as electroretinography and *in vivo* imaging to define the earliest retinal deficits would help to define the role of iron regulation on retinal degeneration. Current animal studies indicate that the major driving factor in retinal degeneration may be iron overload as opposed to the genetic mutation itself having a direct effect, even though many hemochromatosis molecules have been found in the retina (Gnana-Prakasam et al., 2009a). This is because in the genetic modification models retinal changes only manifest at older ages, a stage when iron has had a chance to over-accumulate. Furthermore, treating the *Cp-Heph* (Hadziahmetovic et al., 2011b) and *Hepc* (Song et al., 2014) knock out mouse with an iron-chelator decreased retinal iron levels, oxidative stress and ameliorated retinal degeneration. Given different sub-types of hemochromatosis are due to alterations in a variety of genes further interventional studies are still warranted in other animal models.

## Conclusion

Iron is known to play a critical role in the metabolic activities of the retina particularly the RPE and photoreceptors, but also the inner retina. Studies of ocular manifestations of systemic iron loading remain confined to case reports (Hudson, 1953; Davies et al., 1972; Roth and Foos, 1972; Zerbib et al., 2015; Bellsmith et al., 2020), with only one study on cohort of hemochromatosis patients (Menghini et al., 2018). Few studies have systematically assessed retinal function aside from visual acuity. Cumulative evidence from animal studies, showed that polymorphisms and mutations in iron-modulating genes are associated with progressive retinal iron accumulation and retinal injury.

The exact pathophysiology of retinal abnormalities in systemic iron loading-disorders is still unclear. Improved understanding of the relationship between retinal iron imbalance and retinal pathology is likely to help in the development of therapeutic strategies that can restore retinal iron homeostasis and mitigate neurotoxicity.

## Author Contributions

AS wrote the first draft of the manuscript. CN, BB, and DF contributed to manuscript revision, read, and approved the submitted version. All authors contributed to the article and approved the submitted version.

## Conflict of Interest

The authors declare that this study received funding from an Australian Research Council Linkage grant LP160100126 with funding partners AstraZeneca and Biogen Inc. The funder was not involved in the study design, collection, analysis, interpretation of data, the writing of this article, or the decision to submit it for publication.

## Publisher’s Note

All claims expressed in this article are solely those of the authors and do not necessarily represent those of their affiliated organizations, or those of the publisher, the editors and the reviewers. Any product that may be evaluated in this article, or claim that may be made by its manufacturer, is not guaranteed or endorsed by the publisher.
